# [^18^F]Fluciclovine PET/CT Improves the Clinical Management of Early Recurrence Prostate Cancer Patients

**DOI:** 10.3390/cancers14061461

**Published:** 2022-03-12

**Authors:** Anna Giulia Nappi, Cristina Ferrari, Paolo Mammucci, Dino Rubini, Valentina Lavelli, Angela Sardaro, Antonio Rosario Pisani, Giuseppe Rubini

**Affiliations:** 1 Section of Nuclear Medicine, Interdisciplinary Department of Medicine, University of Bari Aldo Moro, Piazza Giulio Cesare 11, 70124 Bari, Italy; anna.giulia.nappi@gmail.com (A.G.N.); ferrari_cristina@inwind.it (C.F.); paolo.mammucci@outlook.com (P.M.); rubini.dino@libero.it (D.R.); valentina.lavelli@gmail.com (V.L.); apisani71@libero.it (A.R.P.); giuseppe.rubini@uniba.it (G.R.); 2 Section of Radiology and Radiation Oncology, Interdisciplinary Department of Medicine, University of Bari Aldo Moro, Piazza Giulio Cesare 11, 70124 Bari, Italy

**Keywords:** prostate cancer, PET/CT, [^18^F]Fluciclovine, biochemical recurrence

## Abstract

**Simple Summary:**

In the challenge between increasingly sensitive PET radiopharmaceuticals for the evaluation of prostate cancer patient in biochemical relapse, the choice of the most accurate PET tracer must be guided by literature data, but above all tailored to the patient’s profile. In describing our single-center experience, we aimed to identify biochemical and clinical–histological factors to be considered in patient selection and the semiquantitative parameters that can help the interpretation of malignant from benign lesions, in order to optimize the performance of this imaging method. These data in combination with a significant impact on therapeutic decision making can be useful to further validate the [^18^F]Fluciclovine PET/CT clinical application.

**Abstract:**

We investigated the [^18^F]Fluciclovine PET/CT reliability in the early detection of recurrent prostate cancer (PCa) and its impact on therapeutic decision making. We retrospectively analyzed 58 [^18^F]Fluciclovine PET/CT scans performed to identify early PCa recurrence. Detection rate (DR) and semiquantitative analysis were evaluated in relation to biochemical and clinical–histological features. Clinical follow-up data were collected and considered as gold standard to evaluate sensitivity, specificity, accuracy, positive and negative predictive value (PPV, NPV). The impact of [^18^F]Fluciclovine PET/CT on clinical management was also assessed. Overall DR resulted as 66%, while DR was 53%, 28%, and 7% in prostate/bed, lymph nodes, and bone, respectively. DR significantly increased with higher PSA values (*p* = 0.009) and 0.45 ng/mL was identified as the optimal cut-off value. Moreover, SUVmax and SUVmean resulted significant parameters in interpreting malignant from benign findings. [^18^F]Fluciclovine PET/CT reached a sensitivity, specificity, PPV, NPV, and accuracy of 87.10%, 80.00%, 87.10%, 80.00%, and 84.31%, respectively. Therapeutic strategy was changed in 51% of patients. Our results support [^18^F]Fluciclovine PET/CT as a reliable tool for early restaging of PCa patients, especially for local recurrence detection, leading to a significant impact on clinical management. Semiquantitative analysis could improve specificity in interpreting malignant from benign lesions.

## 1. Introduction

Despite the improvement in surgical and radiation techniques for clinically localized prostate cancer (PCa), between 27% and 53% of all patients experience a rising prostate-specific antigen (PSA) level after curative-intent primary treatment [1].

Nowadays, PCa relapse remains a diagnostic and therapeutic dilemma, since conventional imaging, including contrast-enhanced computed tomography (CT), Magnetic Resonance Imaging (MRI), and bone scan, often fail to early detect local tumor recurrence and to differentiate between local and systemic progression, especially at low PSA values [2,3]. Consequently, physicians frequently plan therapy based on clinical and pathological risk factors of disease progression, using standard consensus guidelines, instead of recurrence localization detected on imaging [1,4].

However, there is still no consensus on the optimal imaging modality for detecting small local recurrence in cases of low PSA levels. Molecular imaging is being increasingly applied in the management of PCa patients in PSA relapse and innovative radiopharmaceuticals are being proposed in nuclear medicine scenarios to identify a low tumor burden disease with higher sensitivity at an earlier time.

^18^F/^11^C-Choline is a well-established positron emission tomography (PET) radiopharmaceutical, approved by the Food and Drug Administration (FDA) in 2012 for PCa patients with biochemical recurrence, but with suboptimal detection rate especially in prostate/prostatectomy bed at very low PSA values [5,6].

On the other hand, the scientific community is now focusing on prostate-specific membrane antigen (PSMA), labeled with ^68^Gallium (^68^Ga) and less frequently with ^64^Cu or ^18^F, because of its promising results in this setting of patients, even if the lack of FDA approval limits its wide use in daily clinical practice [3].

[^18^F]Fluciclovine is a synthetic amino acid characterized by a favorable biodistribution and irrelevant urinary interference. The encouraging literature data, with a positivity rate ranging from 56% to 83%, led to FDA approval in May 2016 as a PET radiopharmaceutical for restaging PCa patients in biochemical recurrence following prior treatment [7,8,9,10]. Preliminary studies are confirmed by several recent publications, enrolling cohorts of PCa patients with very low PSA values (≤1 ng/mL), preserving an overall detection rate of 59% [11]. Although impact of pre-scan PSA on the [^18^F]Fluciclovine PET/CT positivity rate is known, literature data are conflicting about which PSA threshold could reliably identify the presence of early recurrent disease [12].

The introduction of this imaging technique into clinical practice was supported also by the LOCATE and FALCON trials [7,9] that, focusing on the impact of [^18^F]Fluciclovine PET/CT on guiding PCa patients’ clinical management, registered a significant change in therapeutic decision making (59% and 63%, respectively). Similarly, in the more recent EMPIRE-1 trial, [^18^F]Fluciclovine significantly changed salvage radiotherapy planning, improving failure-free survival [13]. In addition, the increasing clinical use of this innovative and sensitive imaging technique, allows to distinguish between low-volume metastatic disease, recently defined as oligometastatic disease, and polymetastatic disease, leading to a treatment strategy revision in a significant percentage of PCa patients [14].

Hence, to further validate the clinical utility of [^18^F]Fluciclovine PET/CT and optimize its diagnostic value in early recurrence PCa patients, we aimed to investigate the biochemical and clinical–histological features that could influence [^18^F]Fluciclovine PET/CT performance and the impact of PET findings on clinical management and therapeutic decision making.

## 2. Materials and Methods

### 2.1. Population Characteristics

We conducted a retrospective review of consecutive PCa patients restaged for biochemical recurrence (BCR) with [^18^F]Fluciclovine PET/CT at our institution between September 2019 and September 2021.

The inclusion criteria were: (1) proven PCa treated with radical prostatectomy (RP), with or without adjuvant external beam radiation therapy (EBRT), or with primary radiotherapy (RT); (2) proven BCR with rising PSA levels (PSA > 0.2 ng/mL after RP, PSA ≥ 2 ng/mL above the nadir after primary EBRT); and/or (3) clinical suspicious of disease recurrence (symptomatic patients).

According to these criteria, 58 PCa patients were enrolled in this single-center study and considered for the analysis. During anamnesis, physicians collected clinical information, including primary treatment (PR, RT), histological features (initial Gleason Score, GS and stage), previous imaging investigations, previous and ongoing therapies (androgen deprivation therapy, ADT) as well as previous and current PSA values at the time of the scan. The collected data were used to evaluate PSA kinetics (PSA doubling time, PSAdt <12 months, ≥12 months) and stratify patients according to the new European Association of Urology (EAU) BCR risk groups (low- or high-risk) [15].

All patients gave their informed consent for the scientific use of medical data. The present study was approved by the local Ethics Committee (Prot. n. 0012052|08/02/2022|AOUCPG23|COMET|P).

Population characteristics are summarized in detail in Table 1.

### 2.2. Imaging Protocol and Analysis

[^18^F]Fluciclovine PET/CT was performed according to standard protocols. Patients did not undertake any significant exercise for the day before the exam, fasted for at least 4 h before imaging, and were asked not to void 30–60 min prior to injection [15]. All scans were obtained using a hybrid PET/CT scanner (Discovery 710, GE, General Electrics, Milwaukee, WI, USA). Immediately after the intravenous [^18^F]Fluciclovine administration (370 MBq), the CT scan was performed, followed by the PET scan at 3–5 min post-injection from the mid-thigh to the base of the skull (5–6 bed positions). For clinical reasons, the scan was extended to the top of the skull. A 3D acquisition mode PET scan for the same longitudinal coverage, 2.5 min per bed position, was performed. Coregistered CT parameters were: pitch 0.98, gantry rotation speed of 0.5 s/rot, 120 kVp, and modulated tube current of 140 mA. CT images were used both for image fusion and anatomical localization and for attenuation correction of emission data.

Image analysis was carried out using a dedicated console (AW Server 4.7, General Electrics, Milwaukee, WI, USA). All [^18^F]Fluciclovine PET/CT scans were assessed visually, using Maximum Intensity Projection (MIP), transaxial, sagittal and coronal images, and interpreted in consensus by two nuclear medicine physicians (C.F. and V.L.) with at least 3 years’ experience in [^18^F]Fluciclovine PET/CT reading and awareness of clinical data.

Examinations were considered positive if there was any [^18^F]Fluciclovine uptake visually clearly higher than the surrounding background activity that did not correlate with physiological tracer uptake. Bone marrow uptake of vertebra L3 and abdominal aortic blood pool were used as references for lesions larger than 1 cm longest dimension or lesions smaller than 1 cm longest dimension, respectively [16]. In the case of doubtful lesions, a third nuclear medicine physician (A.G.N.) was consulted, and the final diagnosis was reached in consensus.

### 2.3. Data Analysis

#### 2.3.1. [^18^F]Fluciclovine PET/CT Detection Rate

[^18^F]Fluciclovine PET/CT-positive findings were reported as “detection rate” (DR) since histological confirmation was not available or feasible.

The DR on the per-patient and the per-lesion (prostate/prostate bed, lymph node, bone) analyses were investigated in our cohort in relation to biochemical parameters (PSA value < 0.5, 0.5–1, or >1 ng/mL; PSA dt < 12 or ≥12 months), Gleason Score (GS < 8, GS ≥ 8), EAU BCR risk group (low- and high-risk), the interval time from primary treatment to PSA relapse (TTR), primary treatment (RP alone, RT alone or combined), and ongoing hormonal therapy (yes vs. no). The PSA and TTR cut-off values predictive of a positive and negative scan were evaluated.

#### 2.3.2. Follow-Up Data Analysis

At least 3 months clinical follow-up data were collected for the analysis, which included post-PET PSA values, diagnostic investigations, and start/change treatment. These data were considered as gold standard in the evaluation of sensitivity, specificity, accuracy, positive and negative predictive value (PPV, NPV) of [^18^F]Fluciclovine PET/CT, using the following criteria:

- true positive (TP), patients with evidence of [^18^F]Fluciclovine PET/CT finding(s), confirmed by post-PET anatomical imaging or biopsy examination, and/or biochemical response documented by PSA levels decreasing or stable after therapy or PSA increasing under clinical surveillance;
- true negative (TN), patients with no evidence of [^18^F]Fluciclovine PET/CT finding(s), confirmed on post-PET anatomical imaging, and/or PSA decreasing without any therapy;
- false positive (FP), patients with evidence of [^18^F]Fluciclovine PET/CT finding(s), not confirmed by post-PET anatomical imaging, and PSA decreasing without any therapy;
- false negative (FN), patients with no evidence of [^18^F]Fluciclovine PET/CT finding(s) and positive findings on post-PET anatomical imaging, and/or PSA decreasing after therapy.

Follow-up data regarding therapeutic strategy were used to assess the impact of [^18^F]Fluciclovine PET/CT on therapeutic management of PCa patients enrolled in the study.

#### 2.3.3. [^18^F]Fluciclovine PET/CT Semiquantitative Analysis

All positive findings were analyzed by calculating the following PET-based semi-quantitative and volumetric parameters: maximum Standardized Uptake Value (SUVmax), mean Standardized Uptake Value (SUVmean), Metabolic Tumor Volume (MTV), Total Lesion Activity (TLA, defined as SUVmean × MTV), Tumor-to-Background ratio (T/Bratio), resulting in the ratio of the SUVmax of the lesion to the SUVmean of abdominal aorta or bone marrow as background.

The reference lesion (RL) was identified as the highest [^18^F]Fluciclovine uptake lesion and considered for the per-patient analysis. Per-lesion analysis included the highest uptake lesion of prostate/prostatectomy bed, lymph nodes and bone, respectively.

The different distribution of the semiquantitative PET parameters was evaluated in our sample stratified according to biochemical (PSA level, PSAdt) and clinical–histological features (GS, EAU BCR risk group, TTR, primary and ongoing treatments).

#### 2.3.4. Subpopulation Analysis

A further analysis was conducted dividing our sample into subpopulations, based on PET-disease extent, as follows: exclusively prostate/prostatectomy bed disease or extraprostatic progression, with nodal and/or skeletal involvement. In addition, oligometastatic disease was defined as the presence of one to three lesions regardless of site, while polymetastatic as the presence of four or more lesions [17].

The different distribution of biochemical (PSA level, PSAdt) and clinical–histological (GS, EAU BCR risk group, TTR, primary and ongoing treatments) variables and semiquantitative parameters was evaluated in these subpopulations.

### 2.4. Statistical Analysis

Quantitative variables were expressed as mean with standard deviations (SD) and/or as median with range. Categorical variables were presented with absolute and relative frequencies. The Chi-squared and Fisher exact test were employed to analyze differences in categorical variables: PSA value (<0.5, 0.5–1, >1), PSA dt (<12 months, ≥12 months), GS (<8 vs. ≥8), EAU BCR risk group (low-, high-risk), primary treatment (RP alone, RT alone, combined RP+RT) and ongoing hormonal therapy (yes vs. no). The Mann–Whitney U test and Pearson correlation test were used to compare differences between continuous nonnormally distributed variables (PSA level, TTR, semiquantitative parameters).

The performance of [^18^F]Fluciclovine PET/CT in relation to the continuous variables was assessed by receiving operating characteristic (ROC) curves generated by plotting sensitivity versus 1− specificity. The best cut-off values of continuous variables for predicting between positive and negative [^18^F]Fluciclovine PET/CT scan and for distinguishing between malignant from benign findings were determined using Youden’s index. Statistical significance was assumed for p values less than 0.05.

All statistical analyses were performed using SPSS statistical software, version 28 (IBM Corporation, Armonk, NY, USA).

## 3. Results

### 3.1. [^18^F]Fluciclovine PET/CT Detection Rate

**Figure 1:**
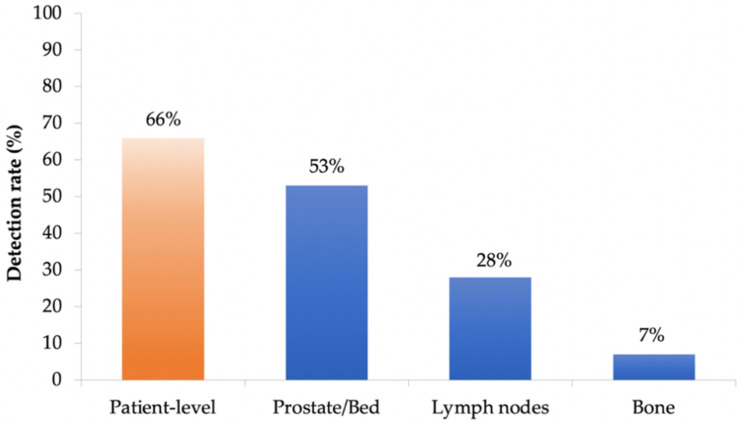
Per-patient and per-lesion detection rate of [^18^F]Fluciclovine PET/CT.

[^18^F]Fluciclovine PET/CT was positive in 66% of patients (38/58), with a DR of 53% (31/58) in prostate/prostatectomy bed, 28% (16/58) in lymph nodes, and 7% (4/58) in bone (Figure 1).

**Figure 2:**
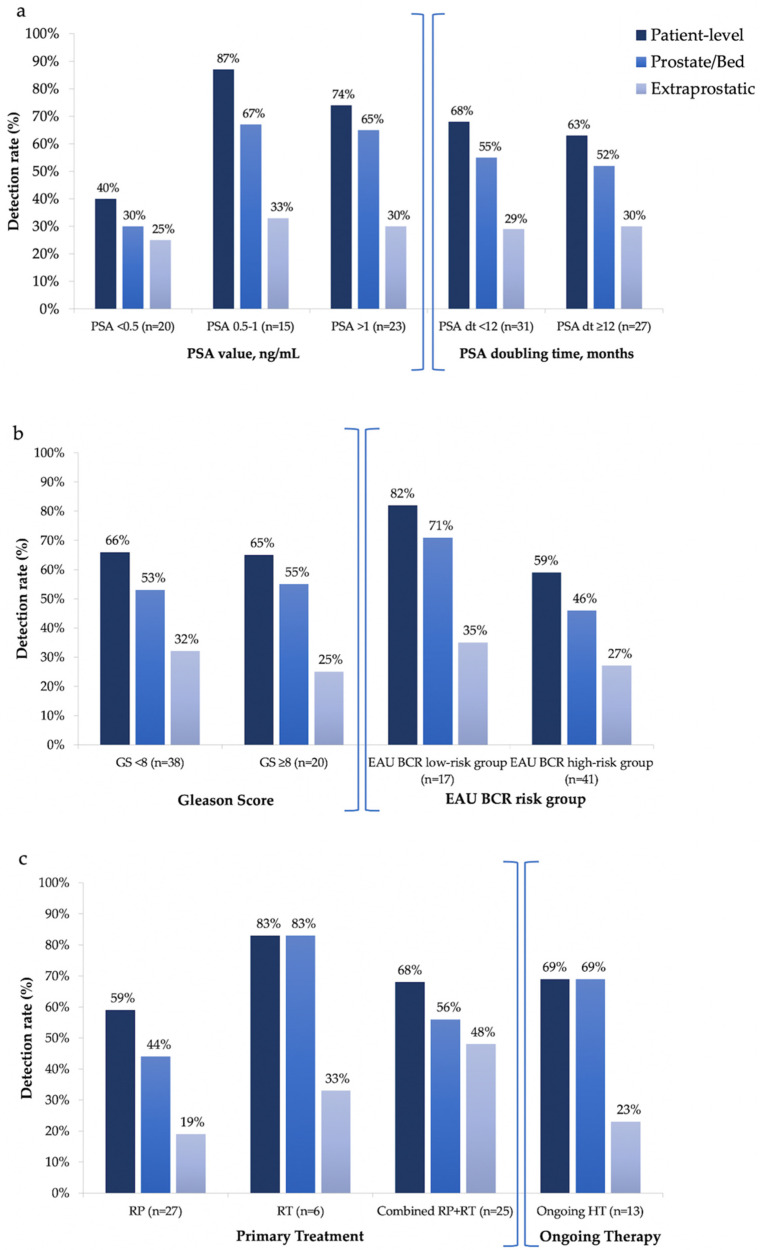
(**a**) Per-patient and per-lesion detection rate of [^18^F]Fluciclovine PET/CT in relation to (**a**) PSA value and PSA doubling time, (**b**) Gleason Score (GS) and European Association of Urology (EAU) biochemical recurrence (BCR) risk groups, and (**c**) primary treatments, including prostatectomy alone (RP), radiotherapy alone (RT) or combined (RP + RT), and ongoing hormonal therapy (HT).

The mean PSA value at the time of the scan was 1.25 ng/mL (median: 0.92 ng/mL; range: 0.05–5.67). [^18^F]Fluciclovine PET/CT-positive patients showed a significantly higher PSA value at the time of the scan than negative ones (*p* = 0.009), with a DR of 40% (8/20) in patients with PSA < 0.5 ng/mL, rising up to 87% (13/15) and 74% (17/23) in PSA 0.5–1.0 ng/mL and PSA > 1.0 ng/mL groups, respectively. Similarly, a significantly higher detection of [^18^F]Fluciclovine PET/CT positive finding(s) resulted in prostate/prostatectomy bed in patients with a higher pre-scan PSA value (*p* = 0.034), with a DR of 30% (6/20) in PSA < 0.5 ng/mL group, rising up to 67% (10/15) and 65% (15/23) in PSA 0.5–1.0 ng/mL and PSA > 1 ng/mL groups, respectively (Figure 2a). No other factor included in the analysis (PSA dt, GS, EAU BCR risk group, ongoing hormonal therapy) resulted as a significant predictor of a positive scan (*p* > 0.05) (Figure 2a–c).

**Figure 3:**
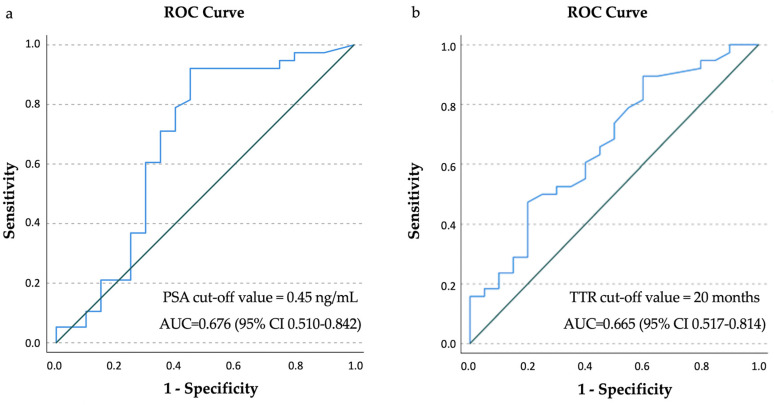
ROC analysis: (**a**) the PSA optimal cut-off value was 0.45 ng/mL with an AUC of 0.676 and (**b**) time from primary treatment to PSA relapse (TTR) optimal cut-off value was 20 months with an AUC of 0.665.

ROC analysis showed a PSA value of 0.45 ng/mL as the optimal cut-off value for predicting positive [^18^F]Fluciclovine PET/CT scan (sensitivity 92%; specificity 55%), with an area under the curve (AUC) of 0.676 (95% CI 0.510–0.842) (Figure 3a).

Similarly, a shorter TTR was significantly associated with a positive [^18^F]Fluciclovine PET/CT scan both on per-patient (*p* = 0.040) and on per-lesion analysis (*p* = 0.030 in prostate/prostatectomy bed), with an optimal cut-off value of 20 months (sensitivity 90%; specificity 40%) and AUC of 0.665 (95% CI 0.517–0.814) (Figure 3b).

### 3.2. Follow-Up Data Analysis

Data regarding clinical follow-up were collected in 88% (51/58) of patients. Patient-level analysis showed true positive findings in 53% (27/51) of patients, true negative in 31% (16/51), false positive in 8% (4/51), and false negative findings in 8% (4/51). Diagnostic performance of [^18^F]Fluciclovine PET/CT was reported in Table 2.

**Figure 4:**
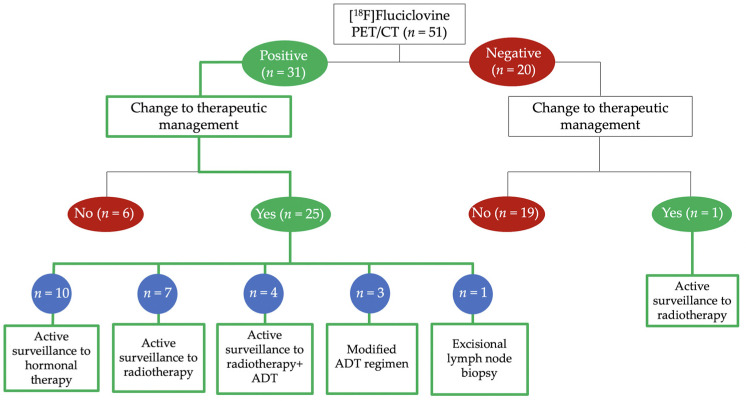
Impact of [^18^F]Fluciclovine PET/CT scan on clinical management of the PCa patients. ADT, androgen deprivation therapy.

**Figure 5:**
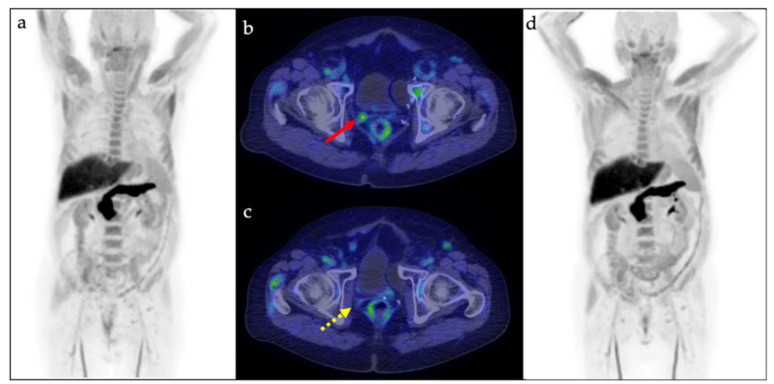
(**a**,**b**) Pre- and (**c**,**d**) post-therapy [^18^F]Fluciclovine PET/CT in a 72 year-old prostate cancer patient in PSA relapse after prostatectomy (2015). Initial stage was pT2cN0 and Gleason Score 7 (4 + 3), classified as high EAU BCR risk group. At the time of the pre-therapy scan, PSA rose to 0.26 with a PSA doubling time of 5.13 months. [^18^F]Fluciclovine PET/CT images ((**a**): Maximum Intensity Projection; (**b**): axial fused PET/CT) showed increased amino acid metabolism in right prostatectomy bed (red arrow). Then, therapeutic strategy was changed, and patient started radiotherapy. After 4 months, post-therapy [^18^F]Fluciclovine PET/CT images ((**c**): axial fused PET/CT; (**d**): Maximum Intensity Projection) documented a metabolic response (yellow dotted arrow), confirmed by the following PSA reduction (0.05 ng/mL).

Clinical follow-up data documented a therapeutic strategy revision after PET in 51% (26/51) of patients, in most cases following a positive scan (96%, 25/26) (Figure 4). The most common pre-scan management was clinical surveillance. The most frequent changes observed were towards a hormonal systemic therapy (38%, 10/26) or radiotherapy (31%, 8/26) (Figure 5). Clinical management was not changed in the remaining 49% (25/51) of patients, more frequently after a negative PET scan (76%, 19/25).

### 3.3. [^18^F]Fluciclovine PET/CT Semiquantitative Analysis

Out of all positive patients (66%, 38/58), RL was localized in prostate/prostatectomy bed in 71% (27/38) of patients, in lymph node in 21% (8/38), and in bone in 8% (3/38), and mean values of the relative semiquantitative parameters were SUVmax_RL_ 4.40, T/Bratio_RL_ 1.59, MTV_RL_ 5.09, TLA_RL_ 14,986.82, and SUVmean_RL_ 2.72.

The per-patient semiquantitative analysis showed no significant difference neither according to biochemical variables nor to clinical–histological ones (*p* > 0.05). However, a trend towards increased SUVmax_RL_ and SUVmean_RL_ with higher PSA levels (especially greater than 1.0 ng/mL) was observed, as presented in Table 3. This trend was further investigated with Pearson correlation, confirming a statistically significant linear correlation between PSA level and SUVmax_RL_ (*p* = 0.006) and SUVmean_RL_ (*p* = 0.003).

Using follow-up data as gold standard, ROC analysis identified SUVmax_RL_ and SUVmean_RL_ optimal cut-off values of 2.05 (AUC = 0.618, 95% CI 0.353–0.883) and 1.75 (AUC = 0.624, 95% CI 0.389–0.860), respectively, in order to help the interpretation of malignant from benign lesions.

### 3.4. Subpopulation Analysis

Out of all PET positive patients, 55% (21/38) showed a disease confined in prostate/prostatectomy bed, while 45% (17/38) developed an extraprostatic disease with or without positivity in prostate bed, of which PET images documented a lymph node and bone involvement in 76% (13/17) and 24% (4/17) of patients, respectively.

Oligometastatic disease was observed in 95% (36/38) of positive scans and polymetastatic disease in the remaining 5% (2/38).

The type of curative-intent treatment showed a significantly difference in the two subpopulations. Particularly, patients treated with combined prostatectomy and radiotherapy developed more likely extraprostatic extension on [^18^F]Fluciclovine PET/CT scan (*p* = 0.005) compared with only local relapse. No other significant differences were found between two subpopulations in relation to other biochemical and clinical–histological factors included in the analysis, as shown in Table 4.

**Figure 6:**
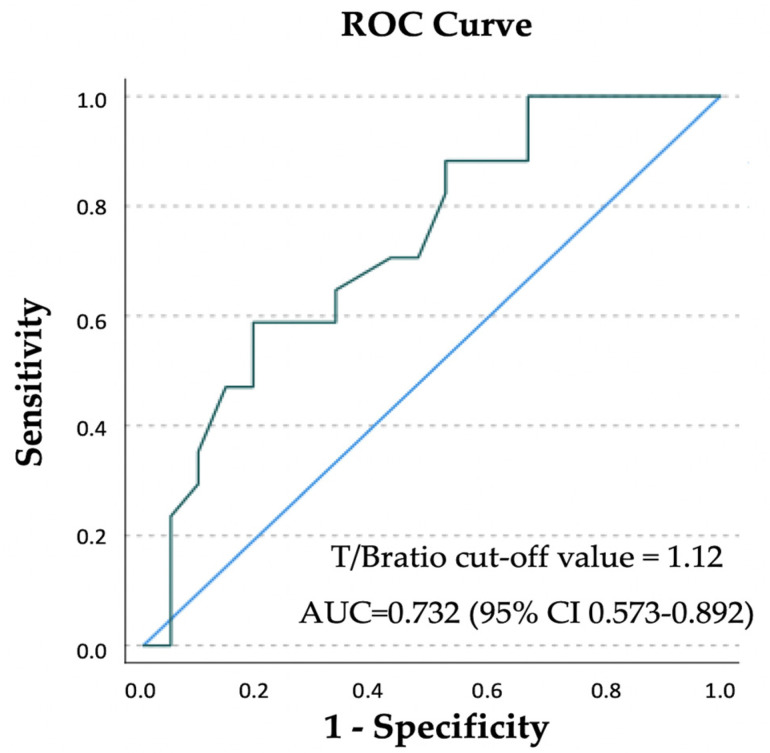
[^18^F]Fluciclovine PET/CT performance in relation to T/Bratio. ROC analysis identified the optimal cut-off value of 1.12 with an AUC of 0.732.

As regards semiquantitative evaluation, a significantly higher T/Bratio_RL_ value (*p* = 0.014) was observed in the extraprostatic disease subpopulation (Table 5). ROC analysis identified a T/Bratio_RL_ optimal cut-off value of 1.12 (sensitivity 88%; specificity 43%) with an AUC of 0.732 (95% CI 0.573–0.892), associated with more likely extraprostatic disease (Figure 6).

**Figure 7:**
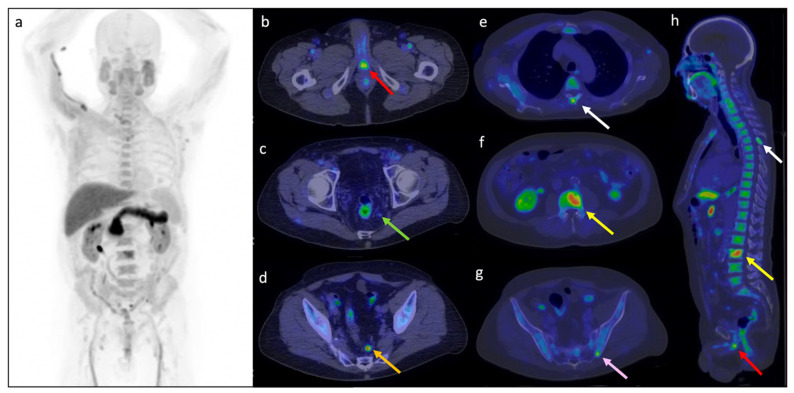
[^18^F]Fluciclovine PET/CT in a 68 year-old prostate cancer patient in PSA relapse after prostatectomy (2011). Initial staging was pT2aN0R0 and Gleason Score 7 (3 + 4), classified as high EAU BCR risk group. At the time of the exam, PSA value rose to 0.76 ng/mL with PSA doubling time of 3.4 months. The images ((**a**): Maximum Intensity Projection; (**b**–**g**): axial fused PET/CT; (**h**): sagittal fused PET/CT) showed increased [^18^F]Fluciclovine uptake in prostatectomy bed with SUVmax 6.4 and T/Bratio 1.6 (red arrow), in left pararectal lymph node with SUVmax 1.9 and T/Bratio 0.95 (green arrow), in the left presacral lymph node with SUVmax 8.0 and T/Bratio 2.0 (orange arrow), in the spinous process of D4 vertebra with SUVmax of 6.3 and T/Bratio 1.5 (white arrow), in L3 vertebra with SUVmax 9.0 and T/Bratio 2.2 (yellow arrow) and left iliac bone with SUVmax 6.0 and T/Bratio 3.0 (pink arrow).

In Figure 7 a representative clinical case of our sample is reported.

## 4. Discussion

Molecular imaging is increasingly integrated in the diagnostic work-up of PCa patients in PSA relapse, yielding higher diagnostic performance compared to conventional imaging and facilitating earlier localization of recurrent disease [18]. For this purpose, new and more sensitive radiopharmaceuticals are challenging in nuclear medicine scenarios, even if there is still no consensus on the optimal imaging modality in the case of low PSA levels.

Current EAU guidelines recommend to perform PSMA-labeled PET/CT for imaging PCa with biochemical recurrence if PSA level is greater than 0.2 ng/mL and if results will influence subsequent treatment decisions; whilst [^18^F]Fluciclovine or Choline-radiolabeled PET/CT are recommended in the case of PSMA-labeled PET/CT is not available and PSA is greater than 1 ng/mL [1]. On the other hand, National Comprehensive Cancer Network guidelines do not indicate a reference PSA value, suggesting [^18^F]Fluciclovine PET/CT after conventional imaging modalities for further evaluation of equivocal findings [19].

Identifying the most suitable radiopharmaceutical based on the PCa patient characteristics, as well as available in clinical practice, would ensure a personalized diagnostic approach for guiding therapeutic decision making. For this purpose, we investigated the biochemical and clinical–histological parameters that could impact on the [^18^F]Fluciclovine PET/CT positivity rate in a cohort of PCa patients with low PSA values in order to optimize its diagnostic performance and patient selection.

The impact of PSA value on [^18^F]Fluciclovine PET/CT DR is already well-known in the literature. A multicenter study enrolling 600 PCa patients demonstrated an overall positivity rate of 67.7% (38.7% in prostate/prostatectomy bed, 32.6% in lymph nodes, 26.2% in extrapelvic sites), and of 41.4% in patients with PSA less than 0.79 ng/mL [20]. In a prospective multicenter study by Scarsbrook et al., an overall DR of 56% was registered in 104 recurrent PCa patients and of 33% among patients with pre-scan PSA less than 0.2 ng/mL, suggesting a good performance also at very low PSA levels [7]. Higher positivity rate (65%) resulted in the Dreyfuss et al. retrospective study with 328 PCa patients, preserving an optimal DR (58%) also among 26 patients with pre-scan PSA value less then 0.2 ng/mL [21]. Consistent with literature, our results confirmed an overall DR of 66% with the pre-scan PSA value as the main predictive parameter of a positive exam, with increasing DR in PSA greater than 0.5 ng/mL (40% in PSA < 0.5 ng/mL; 87% in PSA = 0.5–1 ng/mL; 74% in PSA > 1 ng/mL), especially in prostate bed.

However, data regarding the most reliable PSA threshold for recurrence detection on imaging are conflicting. Several PSA values, variable from 0.3 to 1 ng/mL, are suggested in the literature as a reliable threshold for a [^18^F]Fluciclovine positive scan [22,23]. In our analysis a PSA cut-off value of 0.45 ng/mL was identified as adequate for patient selection.

Most of positive findings were observed in prostate/prostatectomy bed (53%), thanks to irrelevant urinary interference, compared to lymph node (28%) and bone involvement (7%). Differently, the Bulbul et al. study reported a similar prostatic and extraprostatic detection rate of 35% and 37% in patients with PSA less than 1 ng/mL [11].

In relation to cancer biological behavior, a higher [^18^F]Fluciclovine PET/CT DR was documented in more aggressive PCa [24,25], particularly in case of GS≥8, T3-T4 stages, and castration-resistant neoplasm [26]. To the best of our knowledge, this is the first study thar investigated the role of TTR in [^18^F]Fluciclovine PET/CT DR, already evaluated for other radiopharmaceuticals [17]. In our analysis, TTR resulted as the only clinical parameter related to tumor aggressiveness, showing a significant inverse correlation with DR, with an optimal cut-off of 20 months. Differently, statistical significance was not reached for the recently validated EAU BCR risk groups [15], investigated only by Selnæs et al., who reported a lower [^18^F]Fluciclovine PET/MR DR in the EAU low-risk BCR group [27].

Type of primary treatment with curative intention could influence the site of disease recurrence observed on [^18^F]Fluciclovine PET scan. Across all patients enrolled in the FALCON trial, DR was significantly lower in those who underwent prostatectomy (32%) compared with those with an intact prostate (95%), due to prostate/bed findings [18]. Our analysis demonstrated that patients treated with combined surgical and irradiation therapy developed more likely extraprostatic progression (*p* = 0.005). We can speculate that combined therapy reaches a better prostatic disease control rather than extraprostatic one while an inadequate PCa staging performed with poor sensitive imaging methods could cause primary treatment failure with disease recurrence. Furthermore, consistent with preclinical results, our data demonstrated the absence of a negative impact of ADT on [^18^F]Fluciclovine PET/CT positivity rate [28].

According to the EANM procedure guidelines [16], the interpretation of [^18^F]Fluciclovine PET images should be based on the visual analysis. Although in the absence of standardized criteria, the semi-quantitative analysis could be helpful in the interpretation of PET images, especially in doubtful or equivocal cases. Zanoni et al. defined SUVmax as an imaging biomarker predictor of disease progression, being significantly correlated with biological aggressiveness of prostate cancer [29]. Confirming this data, our results demonstrated a statistically significant linear correlation between SUVmax_RL_ (*p* = 0.006) and SUVmean_RL_ (*p* = 0.003) and PSA level.

However, the role of SUVmax appears controversial in differentiating malignant from benign lesions, thus compromising the [^18^F]Fluciclovine PET/CT specificity [29]. In the same study, the measuring of T/Bratio-AORTA and T/Bratio-L3 significantly improved the specificity and sensitivity of this imaging method with the optimal cut-off values of 2.7–3.75 and 1.35–1.55, respectively [29]. In our cohort, SUVmax and SUVmean resulted helpful in interpreting malignant/benign lesions, with optimal cut-off values of 2.05 and 1.75, respectively. In addition, higher T/Bratio values were found in extraprostatic, more advanced, disease. However, further study is needed to validate the role of semiquantitative analysis and identify standardized cut-off values in PET images’ interpretation.

Consistent with the LOCATE [9] and FALCON trials [7], a significant change (51%) in therapeutic strategy was observed after the [^18^F]Fluciclovine PET/CT scan. In the LOCATE and FALCON trials, the most common pre-scan management was salvage radiotherapy. In the LOCATE study, the treatment strategy was frequently changed from salvage radiotherapy or systemic therapy to watchful waiting in [^18^F]Fluciclovine-negative patients (25%), while in positive ones from ADT to radiotherapy (24%), and less commonly the contrary (9%) [9]. In the FALCON trial, salvage radiotherapy was frequently changed to systemic therapy (24%) after a positive scan or similarly to watchful waiting after a negative one (24%) [7]. In our analysis, the most frequent changes were from clinical surveillance to systemic hormone therapy (38%) or radiotherapy (31%) following a positive scan, avoiding an undertreatment.

The clinical management is closely influenced by an accurate imaging definition of the real disease extent and particularly by the emerging importance to differentiate oligometastatic from polymetastatic disease. While current data suggest promising sensitivity of PSMA-based PET radiopharmaceuticals, the growing evidence for the good performance of [^18^F]Fluciclovine at low PSA levels indicates that amino acid tracers should play an important role for the imaging of oligometastatic disease [14,17]. In our cohort, a great percentage (95%) of positive patients was defined as oligometastatic disease, with influence on therapeutic decision making.

Regarding [^18^F]Fluciclovine PET/CT performance in detecting PCa recurrence, literature reports heterogeneous data [30]. A recent meta-analysis registered a pooled sensitivity and specificity of 86.3% and 75.9%, respectively [25]. On the other hand, a lower specificity of 40% was documented in the Schuster et al. analysis with a higher sensitivity of 90.2% [31]. Similar diagnostic performance was found in a larger cohort of 596 PCa patients (sensitivity 88.1%, specificity 32.6%) [20]. Our data confirmed a good [^18^F]Fluciclovine PET/CT sensitivity (87%); however, a higher specificity (80%) emerged in our study. The lack of tissue sampling as gold standard in our analysis could have impacted on this result.

In the nuclear medicine challenge between more sensitive PET radiopharmaceuticals, the trump card remains to ensure an increasingly personalized diagnostic approach to guide a tailored therapeutic choice. Even if the comparison of the different radiopharmaceuticals does not fit the goal of this study, this issue deserves a consideration. In the last 5 years, literature provided comparison data between PET tracers. [^18^F]Fluciclovine demonstrated a greater detection rate compared to ^18^F/^11^C-Choline radiopharmaceuticals, that remains suboptimal especially for low PSA levels [6,32,33]. Conversely, the head-to-head comparisons between [^18^F]Fluciclovine and ^68^Ga-PSMA PET/CT resulted controversial [34]. Pernhtaler et al. registered a more accurate detection of local recurrence of [^18^F]Fluciclovine than 68Ga-PSMA (37.9% vs. 27.6%), especially when located in close relation to the urinary bladder [10]; while, Calais et al. suggested PSMA as the tracer of choice for patients in BCR after radical prostatectomy, since an overall significantly higher detection of PSMA-PET was observed (56% vs. 26%) [35]. On the other hand, it should be noted that limited availability of PSMA-labeled tracer restricted its clinical application, unlike [^18^F]Fluciclovine is more accessible in the US and Europe [6]. Consequently, the best PET target remains a dilemma and it needs to be further investigated. Knowing strengths and weaknesses of each radiopharmaceutical could help to choose the best imaging method, optimized on PCa patient profile, in order to improve the clinical management.

There are some limitations to the present analysis: first, the retrospective nature and the small sample size of this single-center study; second, the lack of tissue sampling as gold standard to assess the diagnostic accuracy of this imaging technique; lastly, the need of long-term follow-up data to investigate patients’ outcomes after PET-induced change in therapeutic strategy.

## 5. Conclusions

[^18^F]Fluciclovine PET/CT is a reliable tool for early restaging PCa patients, with an optimal PSA cut-off value of 0.45 ng/mL to be considered for patient selection. The significant [^18^F]Fluciclovine detection of recurrent lesions (66%) at an earlier time, especially in prostate bed (53%), due to its irrelevant urinary interference, lead to a significant impact on clinical management (51%). Semiquantitative analysis, especially SUVmax and SUVmean, could improve specificity in helping the interpretation of malignant from benign lesions.

## Figures and Tables

**Table 1:** Population characteristics (*n* = 58).

Characteristic	Value
No. patients	58
Age, y	
Mean (SD)	71 ± 7.17
Median (range)	72 (50–83)
Time from primary treatment to BRC, months	
Mean (SD)	60.24 ± 54.09
Median (range)	43 (1–219)
PSA level, ng/mL	
Mean (SD)	1.25 ± 1.18
Median (range)	0.92 (0.05–5.67)
PSA value, *n* (%)	
<0.5 ng/mL	20/58 (34%)
0.5–1 ng/mL	15/58 (26%)
>1 ng/mL	23/58 (40%)
PSA Doubling Time, months	
Mean	52
Median (range)	9 (4.20–2241.10)
PSA Doubling Time, *n* (%)	
<12 months	31/58 (53%)
≥12 months	27/58 (47%)
Gleason Score, *n* (%)	
<8	38/58 (66%)
≥8	20/58 (34%)
EAU BCR Risk Group, *n* (%)	
Low-Risk	17/58 (29%)
High-Risk	41/58 ((71%)
Primary treatment, *n* (%)	
RP	27/58 (47%)
RT	6/58 (10%)
RP + RT	25/58 (43%)
Ongoing hormonal therapy, *n* (%)	
Yes	13/58 (22%)
No	45/58 (78%)

Abbreviations: PSA: Prostate Specific Antigen; EAU: European Association of Urology; BCR: Biochemical recurrence; RP: radical prostatectomy; RT: radiotherapy.

**Table 2:** Diagnostic performance of [^18^F]Fluciclovine PET/CT.

[^18^F]Fluciclovine PET/CT Diagnostic Performance		
	Value	95% CI
Sensitivity	87.10%	70.17% to 96.37%
Specificity	80.00%	56.34% to 94.27%
PPV	87.10%	73.55% to 94.25%
NPV	80.00%	60.96% to 91.11%
Accuracy	84.31%	71.41% to 92.98%

Abbreviations: PPV: Positive Predictive Value; NPV: Negative Predictive Value.

**Table 3:** Per-patient semiquantitative analysis in relation to biochemical and clinical–histological variables.

[^18^F]Fluciclovine PET Parameters	Mean SUVmax_RL_ (Range)	*p*	Mean T/Bratio_RL_ (Range)	*p*	Mean MTV_RL_ (Range)	*p*	Mean TLA_RL_ (Range)	*p*	Mean SUVmean_RL_ (Range)	*p*
PSA Value, ng/mL										
<0.5	3.85	0.149	1.74	0.106	6.44	0.416	16,683.44	0.618	2.35	0.195
	(2.90–5.24)		(0.70–2.60)		(2.03–15.43)		(3392.10–44,146.60)		(1.63–3.34)	
0.5–1	3.93		1.2		5.3		16,111.94		2.51	
	(1.6–9.00)		(0.70–2.20)		(0.66–16.87)		(1089.20–81,840.10)		(0.93–5.90)	
>1	5.03		1.82		4.32		13,529.53		3.05	
	(2.20–12.20)		(0.60–5.23)		(1.75–11.99)		(3708.10–34,779.60)		(1.29–7.90)	
PSA doubling time, months										
<12	5.00	0.45	1.77	0.128	4.58	0.601	15,519.87	0.954	2.88	0.504
	(1.90–12.20)		(0.70–5.23)		(0.66–15.43)		(1089.20–81,840.10)		(1.16–7.90)	
≥12	3.96		1.3		5.51		13,479.49		2.46	
	(1.60–7.90)		(0.60–2.60)		(1.10–16.87)		(1952.90–38,385.20)		(0.93–4.81)	
Gleason Score										
<8	4.49	0.952	1.61	0.627	4.82	0.259	14,511.89	0.3	2.81	0.927
	(2.00–12.20)		(0.60–5.23)		(0.66–16.87)		(1089.20–81,840.10)		(1.20–7.90)	
≥8	4.14		1.54		5.72		16,239		2.51	
	(1.60–7.50)		(0.90–2.90)		(2.24–15.43)		(2079.90–44,146.60)		(0.93–4.30)	
EAU BCR Risk Group										
Low-Risk	4.15	0.917	1.22	0.058	5.1	0.846	12,605.06	0.846	2.5	0.964
	(2.10–7.90)		(0.60–1.80)		(1.10–16.87)		(1952.90–38,385.20)		(1.29–4.81)	
High-Risk	4.58		1.8		5.22		16,861.75		2.82	
	(1.60–12.20)		(0.70–5.23)		(0.66–15.43)		(1089.20–81,840.10)		(0.93–7.90)	
TTR, months										
≤20	5.65	0.982	1.97	0.505	6.49	0.63	22,583.48	0.505	3.65	0.697
	(3.1–12.20)		(0.80–3.80)		(2.37–15.43)		(4384.30–44,146.60)		(1.90–7.90)	
>20	4.25		1.55		4.92		14,093.1		2.62	
	(1.60–9.00)		(0.60–5.23)		(0.66–16.87)		(1089.20–81,840.10)		(0.93–5.90)	
Primary treatment										
RP	4.16	0.835	1.29	0.141	4.1	0.237	10,219.34	0.197	2.53	0.913
	(1.60–7.90)		(0.60–2.20)		(0.66–16.87)		(1089.20–38,385.20)		(0.93–4.81)	
RT	3.82		1.25		8.45		22,677.74		2.59	
	(2.10–5.00)		(0.70–1.79)		(1.10–15.43)		(1952.90–44,146.60)		(1.80–3.40)	
RP + RT	4.69		1.99		4.81		16,444.07		2.88	
	(2.40–12.20)		(0.70–5.23)		(1.75–13.98)		(3392.10–81,840.10)		(1.63–7.90)	
Ongoing hormonal therapy										
Yes	4.73	0.521	1.85	0.1	6.81	0.325	22,207.29	0.124	2.88	0.436
	(1.90–9.00)		(0.70–5.23)		(1.75–15.43)		(4531.70–81,840.10)		(1.16–5.90)	
No	4.28		1.44		3.98		10,744.88		2.67	
	(1.60–12.20)		(0.60–3.80)		(0.66–16.87)		(1089.20–38,385.20)		(0.93–7.90)	

Abbreviations: PET: Positron Emission Tomography; SUVmax: maximum Standardized Uptake Value; T/Bratio: Tumor-to-Background ratio; MTV: Metabolic Tumor Volume; TLA: Total Lesion Activity; SUVmean: mean Standardized Uptake Value; PSA: Prostate Specific Antigen; EAU: European Association of Urology; BCR: biochemical recurrence; TTR: time from primary treatment to PSA relapse; RP: radical prostatectomy; RT: radiotherapy.

**Table 4:** Subpopulations analysis in relation to biochemical and clinical–histological variables.

Characteristic	Overall Positivity (*n* = 38)	Prostate/Bed Disease (*n* = 21)	Extraprostatic Disease (*n* = 17)	*p* Value	Oligometastatic Disease (*n* = 36)	Polymetastatic Disease (*n* = 2)	*p* Value
PSA Value, ng/mL							
Mean (SD)	1.38 ± 1.20	1.35 ± 1.20	1.35 ± 1.23	0.706	1.43 ± 1.21	0.61 ± 0.21	0.182
Median (range)	1 (0.05–5.67)	0.96 (0.26–5.67)	1.00 (0.05–5.23)		1.00 (0.05–5.67)	0.61 (0.46–0.76)	
PSA Value, ng/mL							
<0.5	8/38 (21%)	3/21 (14%)	5/17 (29%)	0.518	7/38 (18%)	1/2 (50%)	0.379
0.5–1	13/38 (34%)	8/21 (38%)	5/17 (29%)		12/36 (33%)	1/2 (50%)	
>1	17/38 (45%)	10/21 (48%)	7/17 (41%)		17/36 (47%)	0/2 (0%)	
PSA doubling time, months							
<12	21/38 (55%)	12/21 (57%)	9/17 (53%)	0.527	19/36 (53%)	2/2 (100%)	0.299
≥12	17/38 (45%)	9/21 (43%)	8/17 (47%)		17/36 (47%)	0/2 (0%)	
Gleason Score							
<8	25/38 (66%)	13/21 (62%)	12/17 (71%)	0.416	24/36 (67%)	1/2 (50%)	0.573
≥8	13/38 (34%)	8/21 (38%)	5/17 (29%)		12/36 (33%)	1/2 (50%)	
EAU BCR Risk Group							
Low-Risk	14/38 (37%)	8/21 (38%)	6/17 (35%)	0.565	14/36 (39%)	0/2 (0%)	0.393
High-Risk	24/38 (63%)	13/21 (62%)	11/17 (65%)		22/36 (61%)	2/2 (100%)	
TTR, months							
Mean (SD)	69.18 ± 56.63	74.76 ± 62.52	62.29 ± 49.40	0.642	71.92 ± 56.86	20 ± 19.80	0.205
Median (range)	53.5 (5–219)	58 (5–219)	38 (6–178)		56.5 (5–219)	20 (6–34)	
RP Alone, *n* (%)							
Yes	16/38 (42%)	11/21 (52%)	5/17 (29%)	0.137	16/36 (44%)	0/2 (0%)	0.329
No	22/38 (58%)	10/21 (48%)	12/17 (71%)		20/36 (56%)	2/2 (100%)	
RT Alone, *n* (%)							
Yes	20/38 (53%)	5/21 (24%)	0/17 (0%)	0.051	5/36 (14%)	0/2 (0%)	0.751
No	18/38 (47%)	16/21 (76%)	17/17 (100%)		31/36 (86%)	2/2 (100%)	
Combined RP + RT, *n* (%)							
Yes	17/38 (45%)	5/21 (24%)	12/17 (71%)	**0.005**	15/36 (42%)	2/2 (100%)	0.193
No	21/38 (55%)	16/21 (76%)	5/17 (29%)		21/36 (58%)	0/2 (0%)	
Ongoing HT, *n* (%)							
Yes	9/38 (24%)	6/21 (29%)	3/17 (18%)	0.346	9/36 (25%)	0/2 (0%)	0.578
No	29/38 (76%)	15/21 (71%)	14/17 (82%)		27/36 (75%)	2/2 (100%)	

Abbreviations: PSA: Prostate Specific Antigen; EAU: European Association of Urology; BCR: Biochemical Recurrence; TTR: time from primary treatment to PSA relapse; RP; radical prostatectomy; RT: radiotherapy; HT: hormonal therapy. Bold: the only statistically significant result.

**Table 5:** Correlation between semiquantitative parameters and subpopulation analysis.

[^18^F]Fluciclovine PET Parameters	Overall Positivity (*n* = 38)	Prostate/Bed Disease (*n* = 21)	Extraprostatic Disease (*n* = 17)	*p* Value	Oligometastatic Disease (*n* = 36)	Polymetastatic Disease (*n* = 2)	*p* Value
SUVmax_RL_							
Mean (SD)	4.37 ± 2.18	4.24 ± 1.80	4.205 ± 2.74		4.31 ± 2.04	6.10 ± 4.10	
Median	4	4.1	3.16	0.862	4.1	6.1	0.597
Range	1.60–12.2	1.9–7.9	1.60–12.20		1.60–12.20	3.20–9.00	
T/Bratio_RL_							
Mean (SD)	1.59 ± 0.91	1.39 ± 0.97	1.785 ± 0.82		1.57 ± 0.92	2.00 ± 0.28	
Median	1.3	1.2	1.75	**0.014**	1.3	2	0.159
Range	0.60–5.23	0.6–5.23	0.70–3.80		0.60–5.23	1.80–2.20	
MTV_RL_							
Mean (SD)	5.15 ± 4.04	4.9 ± 3.67	4.375 ± 4.54		4.91 ± 3.79	8.18 ± 8.21	
Median	3.88	3.88	2.58	1	3.85	8.18	0.774
Range	0.66–16.87	1.1–15.43	0.658–16.87		0.66–16.87	2.37–13.98	
TLA_RL_							
Mean (SD)	15,118.79 ± 15,635.73	12,804.89 ± 10,867.18	14,943.725 ± 21,128.58		13,424.30 ± 10,896.86	43,112.20 ± 54,769.52	
Median	10,024.8	8312.7	5647.6	0.622	10,064.45	43,112.2	0.774
Range	1089.20–81,840.10	1952.90–44,146.60	1089.20–81,840.10		1089.20–44,146.60	4384.30–81,840.10	
SUVmean_RL_							
Mean (SD)	2.76 ± 1.35	2.62 ± 1.04	2.625 ± 1.79		2.66 ± 1.26	3.90 ± 2.83	
Median	2.4	2.6	1.97	0.977	2.5	3.9	0.597
Range	0.93–7.90	1.16–4.81	0.93–7.90		0.93–7.90	1.90–5.90	

Abbreviations: PET: Positron Emission Tomography; SUVmax: maximum Standardized Uptake Value; T/Bratio: Tumor-to-Background ratio; MTV: Metabolic Tumor Volume; TLA: Total Lesion Activity; SUVmean: mean Standardized Uptake Value; SD: standard deviation. Bold: the only statistically significant result.

## Data Availability

The data presented in this study are available on request from the corresponding author.
